# A High-Throughput Memory Compression Architecture for Real-Time 3D Visual Tracking and Quantification on Edge Devices

**DOI:** 10.3390/s26175656

**Published:** 2026-09-05

**Authors:** Yanbo Cheng, Lan Guo, Rui Zhou, Qingguo Zhou, Ling-Huey Li, Mirjana Ivanović, Kuan-Ching Li, Guodong Ye

**Affiliations:** 1 School of Information Science and Engineering, Lanzhou University, Lanzhou 730000, China; chengyb2024@lzu.edu.cn (Y.C.); guol2023@lzu.edu.cn (L.G.); zhouqg@lzu.edu.cn (Q.Z.); 2 Department of Computer Science/DI, University of Salerno, 84084 Fisciano, SA, Italy; lli@unisa.it; 3 Faculty of Sciences, University of Novi Sad, 21000 Novi Sad, Serbia; mira@dmi.uns.ac.rs; 4 Department of Computer Science and Information Engineering, Providence University, Taichung 43301, Taiwan

**Keywords:** runtime memory compression, 3D visual perception, visual and multimedia workloads, edge devices, LZ4, secondary recompression, entropy coding, lossless reconstruction

## Abstract

The deployment of real-time three-dimensional (3D) visual perception, continuous spatial sensing, and graphics-intensive multimedia applications on embedded edge devices introduces substantial runtime memory pressure. During execution, these workloads continuously generate small-granularity memory pages, including image buffers, depth maps, rendering caches, intermediate feature tensors, and media buffers. To reduce memory footprint while preserving low processing overhead, this paper proposes a sequence-aware cascaded compression framework for runtime memory pages generated by visual and multimedia workloads. Instead of following the traditional restore-then-recompress path, the proposed framework directly reuses the intermediate sequence representation produced by LZ4. It parses LZ4 sequences into literal and structured stream sequences and applies stream-specific entropy coding using Huffman and Finite State Entropy (FSE) coding. By avoiding full-page restoration and repeated redundancy analysis, the framework preserves lossless reconstruction semantics while reducing compression overhead. Experimental results on real-world runtime memory pages show that the proposed framework reduces compression processing time by 42.10% on average and by up to 52.22%. It also reduces Central Processing Unit (CPU) time by 20.28% on average and peak memory usage by 29.92% on average. Additional validation on a RISC-V edge platform shows that the compression processing time is reduced by up to 64.36%. A 16 MB point-cloud memory sample derived from 3D scan data further shows a 33.16% reduction in compression time. Functional tests on more than 200K mixed-entropy memory pages demonstrate 100% lossless reconstruction and successful detection of corrupted compressed streams.

## 1. Introduction

With the development of sensor-rich mobile terminals and edge devices, applications such as visual interaction, scene understanding, three-dimensional (3D) visual perception, and multimedia processing have become important workloads in edge-side systems [1,2,3]. During execution, these applications continuously generate a large amount of runtime memory data, including image buffers, depth maps, rendering caches, intermediate feature tensors, and media buffers [1,4,5]. However, mobile terminals and edge devices are typically constrained by limited memory capacity, making it difficult to mitigate the growing runtime memory pressure through straightforward hardware expansion [6]. Therefore, how to effectively compress runtime memory and save memory space while maintaining low processing latency and low system response overhead has become a critical problem for compressed-memory systems [7] and data-intensive visual applications [8,9,10].

Existing runtime memory compression mechanisms usually need to trade off compression speed for effectiveness [7,11,12,13]. One class of methods is represented by fast dictionary-matching compressors such as LZ4 [14]. These methods provide high compression and decompression speed, making them suitable for latency-sensitive system paths. However, since they mainly rely on repeated byte-sequence matching and have limited capability to exploit residual statistical redundancy, their compression effectiveness still has room for improvement. Another class of methods is secondary recompression, where pages are first processed by a fast primary compressor and selected compressed results are then recompressed by algorithms with higher compression effectiveness [7,15]. Such methods can further reduce compressed size, but usually require longer processing time and higher runtime overhead [9,10].

The high overhead introduced by secondary recompression stems largely from the traditional restore-then-recompress approach. In existing mechanisms, the primary compression result is usually treated as an opaque compressed byte stream, making it difficult for the secondary compressor to directly exploit the structural information it already contains. For example, the LZ4 block format represents compressed data using sequences composed of literals, match lengths, and offsets [14], but conventional secondary recompression paths do not directly reuse such sequence-level information. Therefore, before secondary recompression, the system typically needs to restore or reorganize the compressed block into the original page format, after which the secondary compressor scans the page again and rediscovers redundancy. This process not only introduces additional restoration overhead but also underutilizes the sequence-level information extracted during primary compression. In other words, although existing secondary recompression paths can achieve better compression effectiveness, they lack structured coordination between the two compression stages.

To alleviate this contradiction, a more effective approach is to directly reuse the intermediate sequence representation generated by LZ4, rather than falling back to the complete original page for recompression, thereby avoiding repeated processing in the restore-then-recompress path while preserving the size-reduction benefit of secondary recompression. Realizing this idea faces two key challenges. First, recoverable sequence-level information must be extracted from the LZ4-compressed result. Second, appropriate entropy-coding methods must be selected for different parsed data streams so that secondary recompression can efficiently exploit their statistical characteristics.

To address these problems, this work proposes a sequence-aware cascaded compression framework for runtime memory pages generated by visual and multimedia workloads. The framework first uses LZ4 to expose local repetitions in memory pages and generate a recoverable sequence intermediate representation. Then, instead of restoring the complete page, the system directly parses LZ4 sequences and reorganizes their fields into a literal stream and structured sequence streams. According to the statistical characteristics of different streams, the second-stage entropy coder applies Huffman coding [16] to the literal stream and Finite State Entropy (FSE) coding [15,17] to the sequence streams, thereby adapting to the distributions of raw byte symbols and structured state fields, respectively. In this way, the proposed scheme directly reuses the intermediate structure generated by LZ4, avoids full-page restoration and repeated redundancy analysis, and preserves the lossless reconstruction semantics required by runtime memory compression.

Experimental results show that the proposed cascaded compression framework significantly reduces processing overhead while maintaining comparable compression effectiveness. Compared with the restore-then-recompress path, the proposed scheme reduces compression processing time by 42.10% on average and by up to 52.22%. In addition, it reduces Central Processing Unit (CPU) time by 20.28% on average and peak memory usage by 29.92% on average. Functional tests on more than 200K mixed-entropy memory pages show that the proposed scheme achieves 100% lossless reconstruction on the tested pages and can detect corrupted compressed streams in the evaluated scenarios.

## 2. Related Work

### 2.1. Edge Visual and Multimedia Data Compression

Compression has been widely used in edge visual applications to reduce data volume and alleviate resource pressure. Depending on whether reconstruction distortion is allowed, image and multimedia compression methods are commonly divided into lossy and lossless. Lossy compression allows certain information loss in exchange for higher compression density, while lossless compression requires the reconstructed data to be identical to the original data [18]. For lossy compression, Meng et al. used H.264 to compress mobile camera frames before sending them to an edge server for visual processing [1], while AccMPEG reduces transmission bitrate by optimizing video encoding quality while preserving video analytics accuracy [4]. For lossless compression, Walker et al. compared GNU zip (GZIP), LZ4, Blocking, Shuffling and Compression (BLOSC), and Zstandard on microscopy image data [9], and Bernstein and Jakoncic evaluated several fast lossless compressors for diffraction images [10]. These studies demonstrate the importance of compression for edge visual and multimedia workloads, but they mainly operate on files, image frames, video streams, or application-level data representations.

Another related line of research analyzes internal data patterns in visual workloads and uses these patterns to guide subsequent processing. Zhang et al. proposed a video saliency prediction method based on single feature enhancement and temporal recurrence, where salient visual features are enhanced and temporal evolution across frames is modeled to improve video-level saliency prediction [19]. Linardos et al. further studied simple and complex temporal recurrence mechanisms for video saliency prediction, showing that recurrent temporal modeling can help capture video saliency patterns [20]. Although these studies are designed for visual prediction rather than compression, they are conceptually relevant to this work because they show that visual and multimedia data should not always be treated as opaque inputs. Instead, their internal structures and recurring patterns can be extracted and reused to improve system behavior.

Nevertheless, existing visual compression methods and pattern-analysis studies only partially address the requirements of runtime memory compression. Visual compression studies mainly optimize transmission bandwidth, storage footprint, or processing efficiency for frames, files, and streams, rather than page-level runtime memory data. Pattern-aware visual analysis studies exploit spatial or temporal structures for visual prediction [21,22], but they do not use these patterns to reduce memory-compression overhead. In contrast, visual applications also generate a large number of runtime memory pages during execution, including image buffers, rendering caches, media buffers, and intermediate processing data. These pages are located on the system execution path, where compression is constrained by page granularity, lossless recovery, low latency, and bounded runtime overhead [11,23]. Therefore, this work focuses on exploiting the sequence-level structure exposed by LZ4 within runtime memory pages, so that internal data patterns can be reused to support efficient secondary recompression.

### 2.2. Compression Algorithms for Runtime Memory Data

Runtime memory compression places stronger emphasis on low latency, high throughput, lossless recovery, and bounded memory overhead than conventional file compression [7,11,12,13]. Dictionary matching and entropy coding are two representative approaches for runtime memory compression. Dictionary-matching methods, such as LZ77, LZ4, LZO, remove redundancy by identifying repeated byte sequences [14,24,25]. These methods are simple and fast, making them suitable for latency-sensitive paths. Entropy-coding methods, such as Huffman coding, arithmetic coding, Asymmetric Numeral Systems (ANS), and FSE, compress data according to symbol frequencies or probability distributions [15,16,17,26]. Compared with dictionary matching, entropy coding can exploit statistical redundancy more effectively but usually requires symbol modeling, coding table construction, and additional data organization. Therefore, a single compression algorithm often needs to trade off compression speed against compression density, and different algorithms offer different trade-offs among compressed size, compression throughput, decompression latency, and memory overhead [27,28,29,30,31,32].

To balance these tradeoffs, secondary recompression has been introduced as a two-stage compression strategy in compressed-memory systems. For example, Linux zram supports a primary compressor and multiple secondary compressors, allowing selected pages to be recompressed after primary compression to improve compression density [7]. In this strategy, a fast primary compressor first processes memory pages to meet latency requirements, and selected compressed pages are then processed by a denser secondary compressor to improve compression effectiveness further. However, existing mechanisms usually organize the two compression stages as a loosely coupled process. The primary compressor’s output is not treated as an intermediate representation that the secondary compressor can directly exploit; instead, it must be restored or reorganized before secondary recompression. As a result, the structural information already captured by the primary compression stage is difficult to explicitly reuse in the subsequent stage. How to coordinate dictionary-based primary compression and entropy-based secondary recompression for fine-grained runtime memory pages remains insufficiently explored.

## 3. Motivation and Problem Analysis

### 3.1. Entropy Characteristics of Runtime Memory Pages

Runtime memory pages generated by visual and multimedia applications exhibit heterogeneous entropy characteristics. Page entropy reflects the randomness of byte contents and can indicate potential compressibility [33]. Low-entropy pages usually contain more regular or repeated byte patterns and are suitable for fast dictionary-matching compressors. Medium-entropy pages may still retain statistical redundancy that can be further exploited by entropy-coding methods. High-entropy pages are closer to random distributions and usually provide limited compression opportunities.

We use byte-level entropy to divide 4-kilobyte pages into three groups. Since byte entropy ranges from 0 to 8 bits per byte, pages with H<2.0 contain less than one quarter of the maximum entropy and usually show highly repetitive patterns. Pages with H>6.5 are close to the random-like range, where the practical compression margin is limited by block granularity and coding overhead. The remaining interval, 2.0≤H≤6.5, is treated as the medium-entropy region, where residual statistical redundancy may still be exploitable after LZ4 compression [33,34].

**Figure 1:**
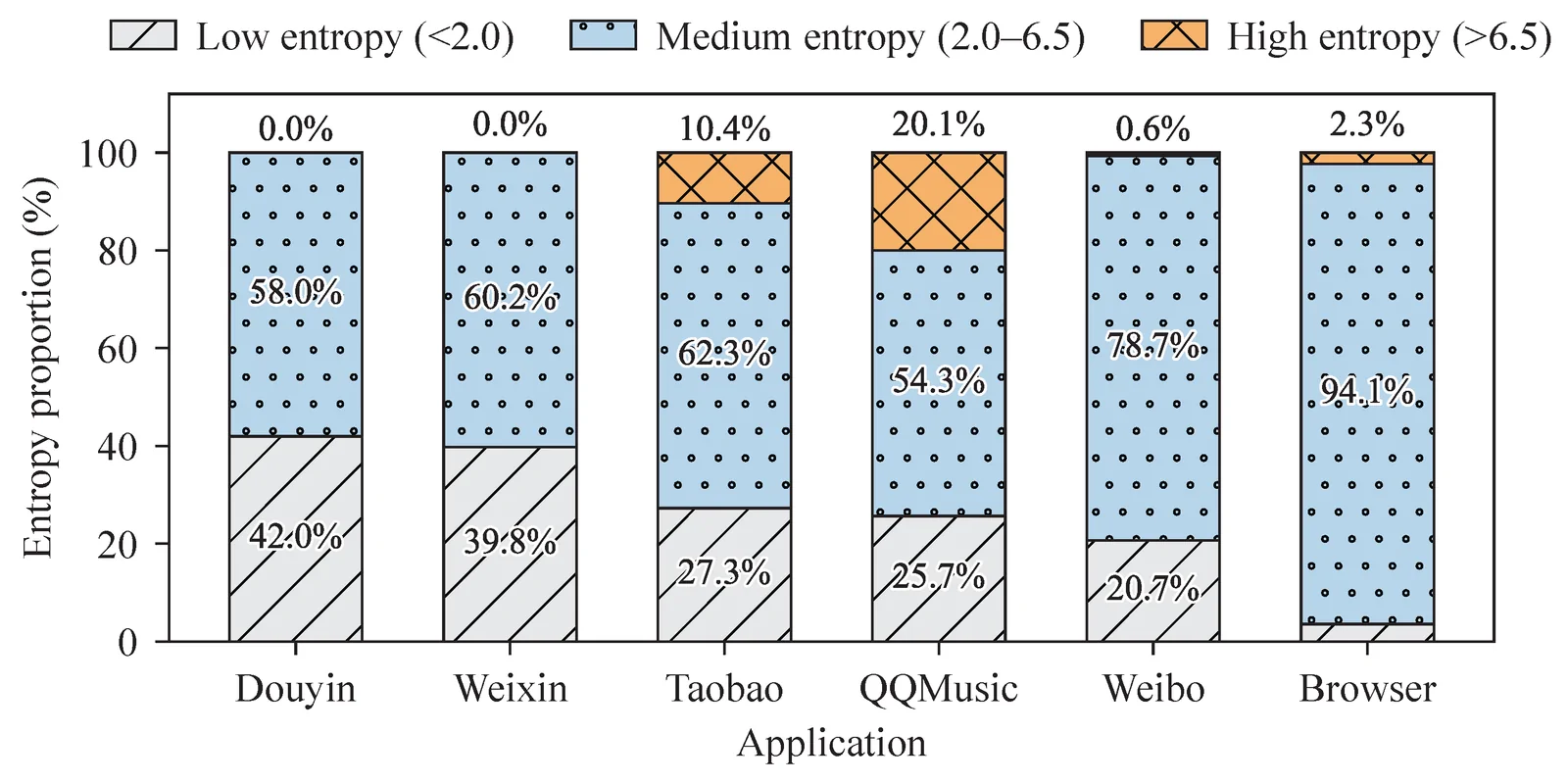
Entropy distribution of runtime memory pages.

Figure 1 shows the entropy distribution of 4-kilobyte runtime memory pages collected from a real mobile terminal during the execution of representative visual and multimedia applications. Douyin and Weixin contain relatively high proportions of low-entropy pages, reaching 42.0% and 39.8%, respectively. Taobao and QQMusic contain higher proportions of high-entropy pages, reaching 10.4% and 20.1%, respectively. The browser is dominated by medium-entropy pages, accounting for 94.1%. These results indicate that runtime memory pages generated by visual and multimedia workloads do not follow a uniform redundancy pattern, and a single compression strategy is unlikely to fully adapt to the heterogeneous characteristics of different pages.

**Figure 2:**
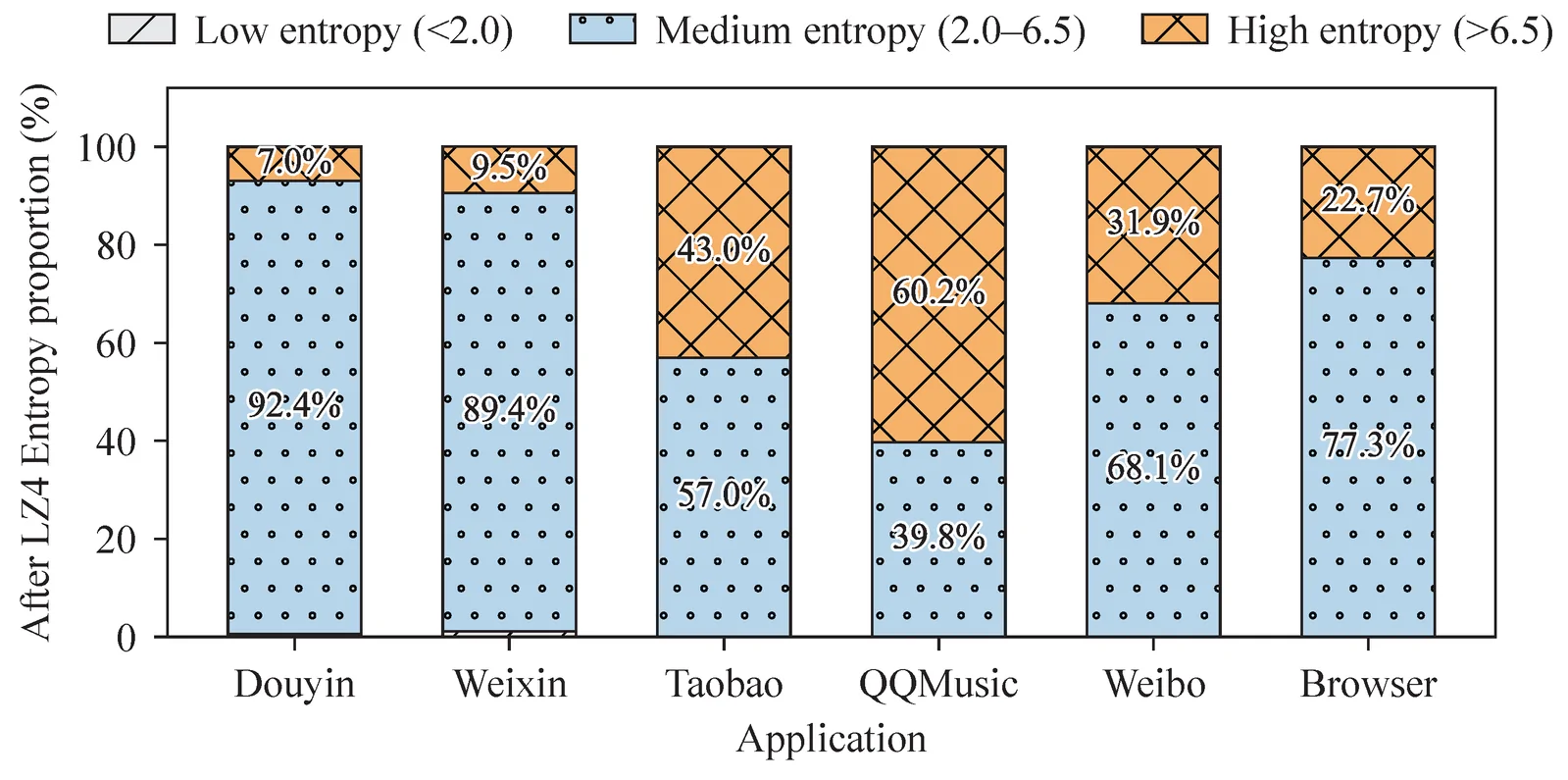
Entropy distribution of LZ4-compressed pages.

Figure 2 further shows the entropy distribution after primary LZ4 compression. A large amount of compressed data remains in the medium-entropy range. Specifically, the medium-entropy proportions for Douyin and Weixin are 92.4% and 89.4%, respectively, while those for Browser and Weibo are 77.3% and 68.1%, respectively. Taobao and QQMusic also retain 57.0% and 39.8% medium-entropy data, respectively, indicating that LZ4 effectively removes explicit repeated byte patterns, but its compressed output may still contain residual statistical redundancy.

Therefore, primary LZ4 compression alone cannot fully exploit the compression potential of runtime memory pages. The large amount of medium-entropy data remaining after LZ4 compression motivates secondary recompression to further reduce residual redundancy.

### 3.2. Cost of Existing Recompression Paths

The restore-then-recompress path can further reduce the compressed size of 4-kilobyte runtime memory pages, but it introduces an additional restoration stage before secondary recompression. Since secondary compressors usually trade longer processing times for higher compression effectiveness, increased compression latency is expected. The more critical issue is that data already processed by the primary compressor must be restored or reorganized before being processed again by the secondary compressor, which adds additional data transformation and repeated analysis overhead.

**Figure 3:**
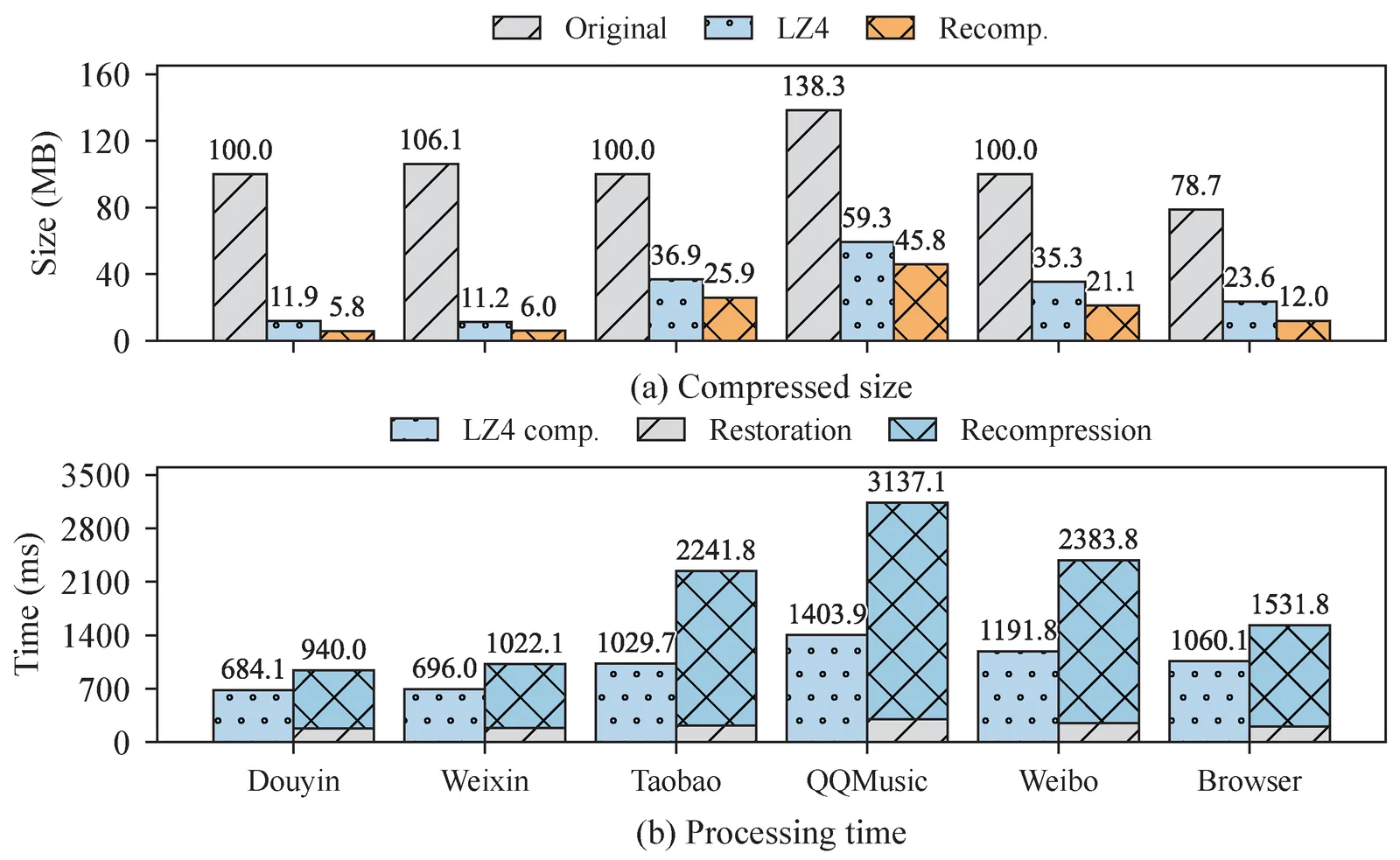
Recompression cost for runtime memory pages. (**a**) Compressed size before and after LZ4 compression and recompression. (**b**) Processing time of LZ4 compression and the recompression path, including restoration and secondary recompression.

Figure 3 shows the compressed size and processing time of the restore-then-recompress path across different applications. As shown in Figure 3a, secondary recompression further reduces the compressed size compared with primary LZ4 compression. For example, the compressed size of Douyin decreases from 11.9 MB after LZ4 compression to 5.8 MB after secondary recompression, while that of QQMusic decreases from 59.3 MB to 45.8 MB. However, Figure 3b shows that this size reduction comes with additional restoration costs. For Douyin, the restoration stage takes an additional 180.8 ms before the 759.3 ms secondary recompression stage. For QQMusic, restoration takes an additional 303.2 ms before the 2834.0 ms secondary recompression stage.

These results indicate that the restore-then-recompress path contains avoidable restoration overhead between primary compression and secondary recompression. Therefore, a compression framework better suited to runtime memory scenarios should preserve the major compression benefit of secondary recompression while avoiding the intermediate restoration step whenever possible.

## 4. Materials and Methods

**Figure 4:**
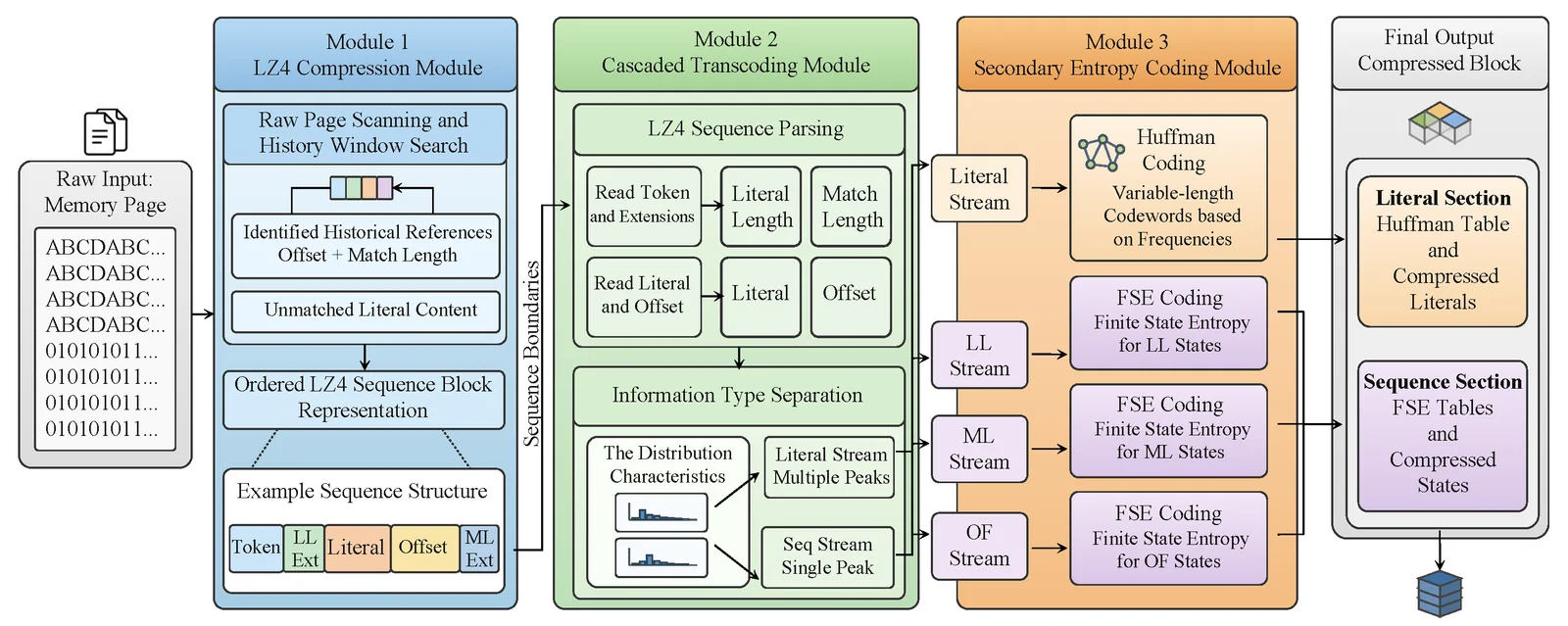
Overview of the proposed framework.

To reduce the compressed size of runtime memory pages while preserving low processing overhead, we design a sequence-aware cascaded compression framework that integrates fast LZ4 matching with secondary entropy coding. As illustrated in Figure 4, the framework starts from a runtime memory page, extracts sequence-level structure through front-end compression and transcoding, and finally produces a recoverable compressed block.

The framework contains three main modules. The LZ4 compression module exposes local repetition in the input page and generates an intermediate LZ4 sequence representation. The cascaded transcoding module parses these LZ4 sequences and separates their fields into a literal stream and structured sequence streams. The secondary entropy-coding module further compresses the two streams using Huffman coding and FSE coding, respectively, and writes the encoded results to the literal and sequence sections of the final compressed block.

### 4.1. LZ4 Compression Module

The LZ4 compression module is designed to make local repetition within runtime memory pages explicit, thereby transforming an originally unstructured page-level byte array into an intermediate representation with clear LZ4 sequence boundaries. Positioned at the front end of the cascaded compression pipeline, this module aims to identify easily matchable repeated fragments within a page with low processing overhead. Specifically, LZ4 sequentially scans the input page and searches a history window for byte sequences identical to the current position. Once a valid match is found, the repeated bytes are no longer stored explicitly; instead, they are represented by a historical reference consisting of a backward offset and a match length. Segments that cannot be matched are preserved as literal content. After this process, the input page is organized into multiple LZ4 sequences, each containing both unmatched literals and reusable historical references. This representation enables the subsequent transcoding module to operate directly on the first-stage compression result, avoiding the additional overhead caused by fully restoring the original page and performing repeated matching in the restore-then-recompress path.

This front-end processing strategy targets the page-level redundancy characteristics discussed in the motivational analysis. Visual perception and multimedia applications continuously generate rendering caches, media buffers, intermediate feature data, and similar runtime content. These data are usually not completely random; instead, they retain varying degrees of local repetition and regularity within memory pages. As shown in Figure 1, the entropy distribution of the collected runtime memory pages spans a wide range, and a portion of pages or page fragments exhibit clear low-entropy characteristics, indicating the existence of repeated byte patterns that can be efficiently captured by fast dictionary matching. Therefore, introducing LZ4 at the front end of the cascaded compression pipeline allows these explicit repetitions to be first converted into parseable historical references, while the unmatched content is retained as literals, providing a structured basis for the subsequent transcoding module to further separate and reorganize LZ4 sequence information.

The cascaded transcoding module takes the LZ4 sequence representation produced by the LZ4 compression module and converts it from the native LZ4 compressed format into a logical data organization that the secondary entropy-coding module can directly consume. Rather than treating the LZ4 output as an opaque compressed byte stream, this module parses the semantic fields embedded in each LZ4 sequence, including literal content, literal length, match length, and offset, and reorganizes them into a literal stream and structured sequence streams according to their information types.

### 4.2. Cascaded Transcoding Module

#### 4.2.1. LZ4 Sequence Parsing

LZ4 sequence parsing is the first step of the transcoding module. Its purpose is to extract the semantic fields required for subsequent transcoding from each compressed LZ4 sequence. The module does not fully decompress the page. Instead, it only reads the control information and literal content within each sequence and decomposes them into four fields: literal length, literal content, match length, and offset.

An LZ4 sequence typically consists of a token, optional length extension fields, literals, an offset, and an optional match-length extension field [14]. The token acts as the control field: its high four bits record the base value of the literal length, while its low four bits record the base value of the match length. When either the literal length or the match length exceeds the range directly represented by the token, the corresponding extension field stores the additional length. The literals field contains the original bytes not covered by historical matches, and the offset field indicates the backward distance to the referenced position in the history window, following the basic dictionary-matching principle of Lempel-Ziv compression [24].

Based on this format, the transcoding module parses the compressed block in sequence order. It first reads the token and the possible length-extension fields to recover the actual literal length and the match length. It then extracts the corresponding literals based on the recovered literal length and reads the offset field when the current sequence matches. Through this procedure, the compact binary representation originally designed for fast LZ4 decompression is converted into semantic fields for subsequent entropy coding.

**Figure 5:**
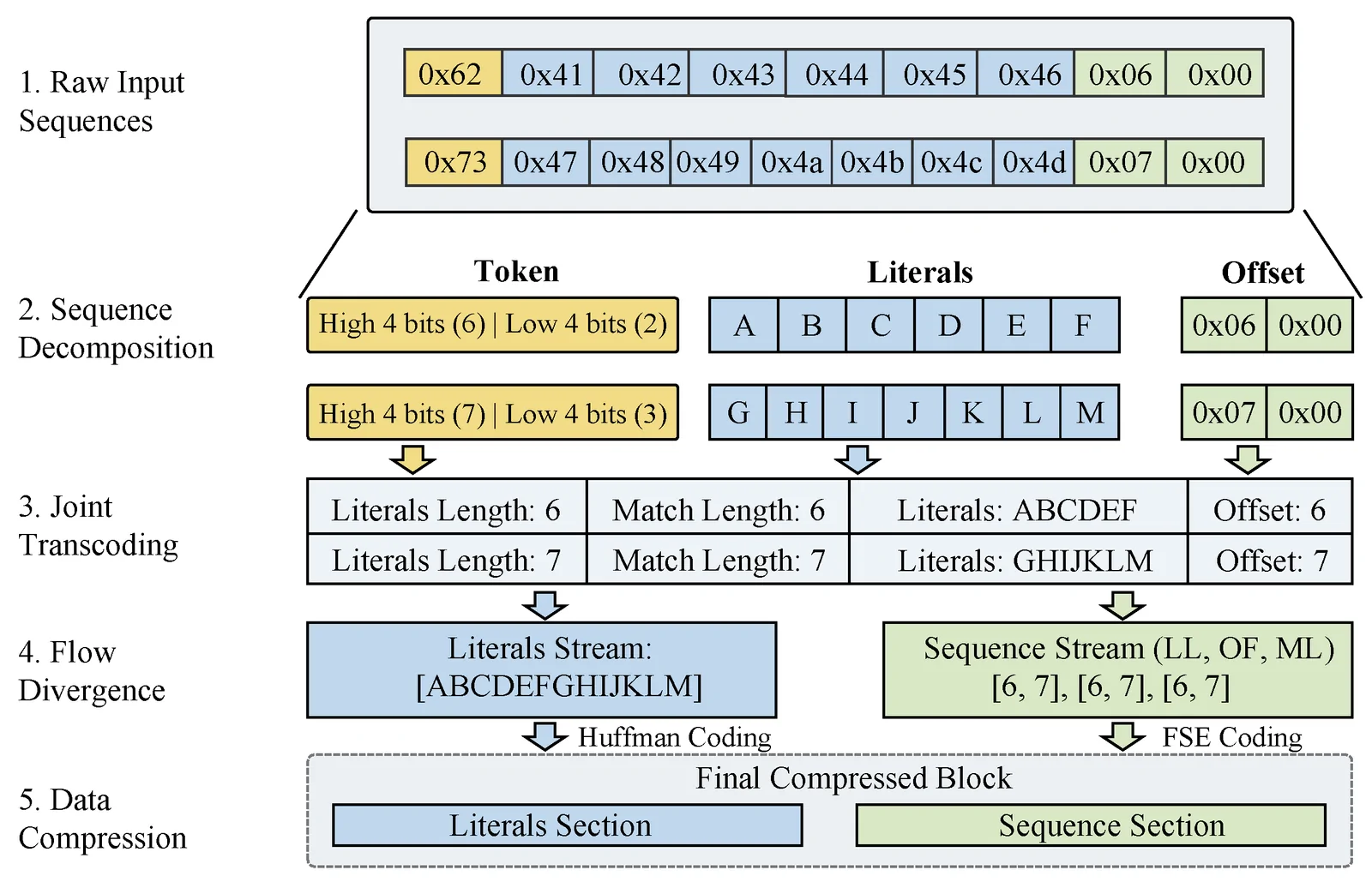
Representative workflow from LZ4 sequence parsing to secondary entropy coding.

The first three parts of Figure 5 present a representative example of field-level parsing for LZ4 sequences in the transcoding module. In this example, the compact byte-oriented input sequences are decomposed into tokens, literals, and offset fields, from which the literal length, match length, literal content, and backward offset are extracted. The parsed fields are then reorganized into a structured representation for subsequent entropy coding without fully reconstructing the original page.

Through the above procedure, the transcoding module completes field-level decomposition of the LZ4 sequence. The extracted fields are then reorganized by information type to form the input representation for the secondary entropy-coding module.

#### 4.2.2. Information Type Separation

After LZ4 sequence parsing, the transcoding module separates the parsed fields by information type and organizes them into two logical data streams. The first is the literal stream, formed by concatenating the literal fields from all LZ4 sequences. The second is the structured sequence streams, consisting of the literal-length (LL) stream, match-length (ML) stream, and offset (OF) stream. This step converts the interleaved fields in the LZ4 format into data streams that can be independently processed by the subsequent coding module.

This separation is motivated by the different statistical distributions of literal bytes and the structured fields LL, ML, and OF. Entropy coding benefits from non-uniform symbol frequencies [34], whereas mixing heterogeneous fields into a single symbol space may weaken their individual frequency characteristics. Literal bytes correspond to raw byte values and span the full byte range (0–255); their distribution is not necessarily uniform but often appears as multiple local peaks across the byte space. In contrast, LL, ML, and OF are structured state information generated during the LZ4 matching process, and their values are more likely to concentrate in a small number of high-frequency regions. Separating these two types of information, therefore, allows the subsequent coding stage to exploit statistical redundancy in both byte content and structured states more effectively.

**Figure 6:**
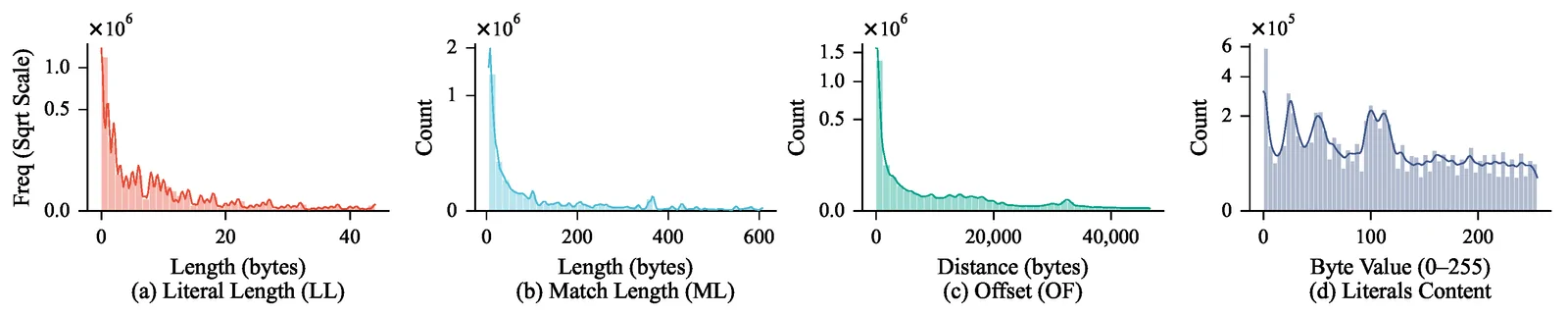
Frequency and density distributions of the parsed LZ4 fields from Douyin memory pages, including literal length (LL), match length (ML), offset (OF), and literal byte values.

Figure 6 validates this difference using Douyin memory pages as an example. LL, ML, and OF exhibit clear head-concentrated and long-tailed distributions. LL is concentrated in short-length regions, ML shows high-frequency peaks in the short-match range, and OF decreases as the reference distance increases. These results indicate that the structured fields are dominated by a limited number of frequent states, making them suitable for state-stream organization.

In contrast, Figure 6d shows that literal bytes follow a multi-peak distribution across the 0–255 byte space, rather than concentrating around a few length or distance states. Therefore, the transcoding module keeps literal bytes as an independent literal stream and organizes LL, ML, and OF as separate sequence streams. The resulting literal stream, LL stream, ML stream, and OF stream provide separate inputs for the subsequent secondary entropy-coding stage.

After this separation, the transcoding module outputs a literal stream and a set of sequence streams, including the LL, ML, and OF streams. These streams preserve the differences among LZ4 fields and provide separated inputs for the subsequent secondary entropy-coding stage.

### 4.3. Secondary Entropy-Coding Module

The secondary entropy-coding module receives the literal stream and sequence streams generated after field reorganization and encodes them through separate entropy-coding paths. As illustrated in Parts 4–5 of Figure 5, literal bytes are directed to a literal-oriented stream, while LL, ML, and OF fields are organized as structure-oriented sequence streams. Since the literal stream exhibits a multi-peak distribution over the 0–255 byte space, whereas the sequence streams are dominated by a few high-frequency states with a long tail, Huffman coding is applied to the former, and FSE coding is applied to the latter.

For the literal stream, Huffman coding is used to generate the literal section. The symbols in this stream are concrete byte values. Although their distribution is non-uniform, it appears as multiple local high-frequency peaks across the byte space. Huffman coding assigns variable-length codewords according to symbol frequencies, giving shorter codewords to frequent symbols and longer codewords to infrequent symbols [16,34]. Therefore, it is suitable for compressing this multi-peak byte distribution. After encoding, the Huffman table description and the compressed literal bitstream are written into the literal section, which is used to restore the unmatched original bytes during decoding.

For the sequence streams, FSE coding is used to generate the sequence section. LL, ML, and OF are structured state symbols rather than raw byte values. Their values do not span the full byte space but concentrate around a small number of frequent states with a low-frequency tail. FSE is a table-based entropy coding method derived from asymmetric numeral systems and can exploit probability differences among symbols to reduce the average coding length [15,17]. This property makes it suitable for modeling structured state streams. To preserve the distribution characteristics of each stream, this module builds independent FSE representations for the LL, ML, and OF streams. After encoding, the FSE table descriptions and the compressed state bitstreams are written into the sequence section.

Finally, the secondary entropy-coding module outputs a compressed block consisting of the literal and sequence sections. The former stores the Huffman-coded literal content, while the latter stores the FSE-coded LL, ML, and OF state information. This packaging format maintains LZ4 sequence semantics while further compressing the two reorganized data streams.

## 5. Experimental Results

### 5.1. Experimental Setup

The main experiments were conducted on a Raspberry Pi 4 Model B with 4 GB memory, which represents a typical resource-constrained edge device. To further examine whether the observed performance trend depends on a single hardware platform, we also conducted a cross-architecture validation on a StarFive VisionFive 2 RISC-V edge platform. On each platform, the proposed sequence-aware cascaded compression framework and the baseline recompression path were evaluated under the same experimental setting to ensure a fair comparison. The main hardware configurations are summarized in Table 1.

### 5.2. Compression Size–Latency Trade-Off

**Figure 7:**
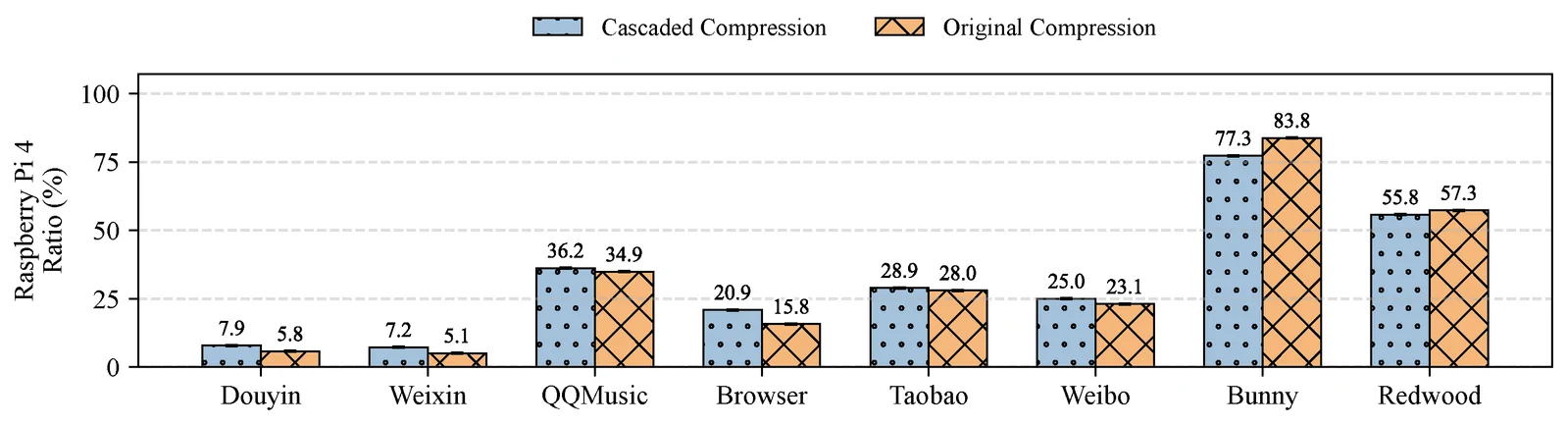
Comparison of overall compressed-size ratio.

**Figure 8:**
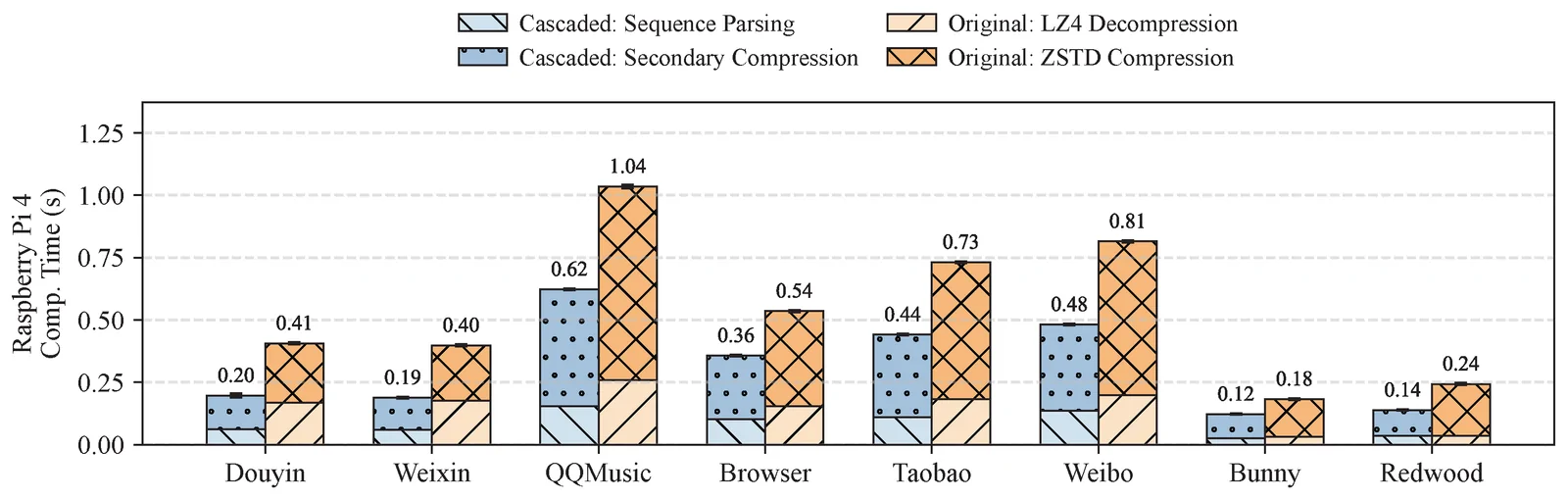
Breakdown of compression processing time.

Figure 7 and Figure 8 compare the proposed framework with the conventional restore-then-recompress path in terms of compressed-size ratio and compression latency. The proposed framework prioritizes compression latency over compression density. Instead of reconstructing full pages and recompressing them from scratch, it reuses the sequence-level representation generated by the LZ4 front end to reduce second-stage compression cost. The proposed framework produces higher compressed-size ratios for the six application workloads but lower ratios for the two 3D datasets, with an average increase of 0.70 percentage points across all eight datasets. The largest gap is observed in the Browser workload, where the difference reaches 5.15 percentage points. For 4-kilobyte runtime memory pages, these differences correspond to an average additional storage cost of approximately 29 bytes per page and up to approximately 211 bytes per page. Equivalently, for every 1 GiB of original page data, the average additional compressed storage is approximately 7.2 MiB, with the largest increase of approximately 52.7 MiB.

In contrast, the proposed framework significantly reduces compression processing time. On average, compression latency is reduced by 42.10% compared with the restore-then-recompress path, with the largest reductions observed in the Douyin and Weixin workloads, where latency decreases by 52.22%. The timing results remain stable across repeated runs, with relative variation typically below 1%. These results show that the proposed framework substantially reduces the processing overhead of restore-then-recompress, while incurring a moderate increase in compressed size.

The latency breakdown in Figure 8 further explains the source of this improvement. The proposed framework directly reuses the LZ4 sequence representation, and its processing time consists of sequence transcoding and compression of the transcoded representation. By contrast, the restore-then-recompress path first restores the LZ4-compressed block into the original page and then recompresses the reconstructed page from scratch. Sequence transcoding is substantially faster than LZ4 decompression, reducing preprocessing time by 35.60% on average and by up to 65.16% in the Weixin workload.

After excluding preprocessing overhead, the subsequent compression stage of the proposed framework remains significantly faster, with an average reduction of 41.92% and a maximum reduction of 50.62%. This indicates that the performance gain does not come merely from avoiding full-page reconstruction. More importantly, by reusing the sequence-level structure already exposed by the LZ4 front end, the proposed framework avoids rediscovering local redundancy from restored raw pages, allowing the entropy-coding stage to operate on a more compact and structured data stream.

Overall, the proposed framework exploits sequence-level redundancy exposed by the primary LZ4 compression stage and eliminates both full-page restoration and repeated redundancy analysis required by restore-then-recompress. Therefore, with a moderate increase in compressed size, the proposed sequence-aware cascade compression framework consistently reduces second-stage compression latency. These results demonstrate the effectiveness of the proposed runtime memory-page compression design for latency-sensitive and resource-constrained online memory-management scenarios.

### 5.3. Compression-Side Resource Overhead

**Figure 9:**
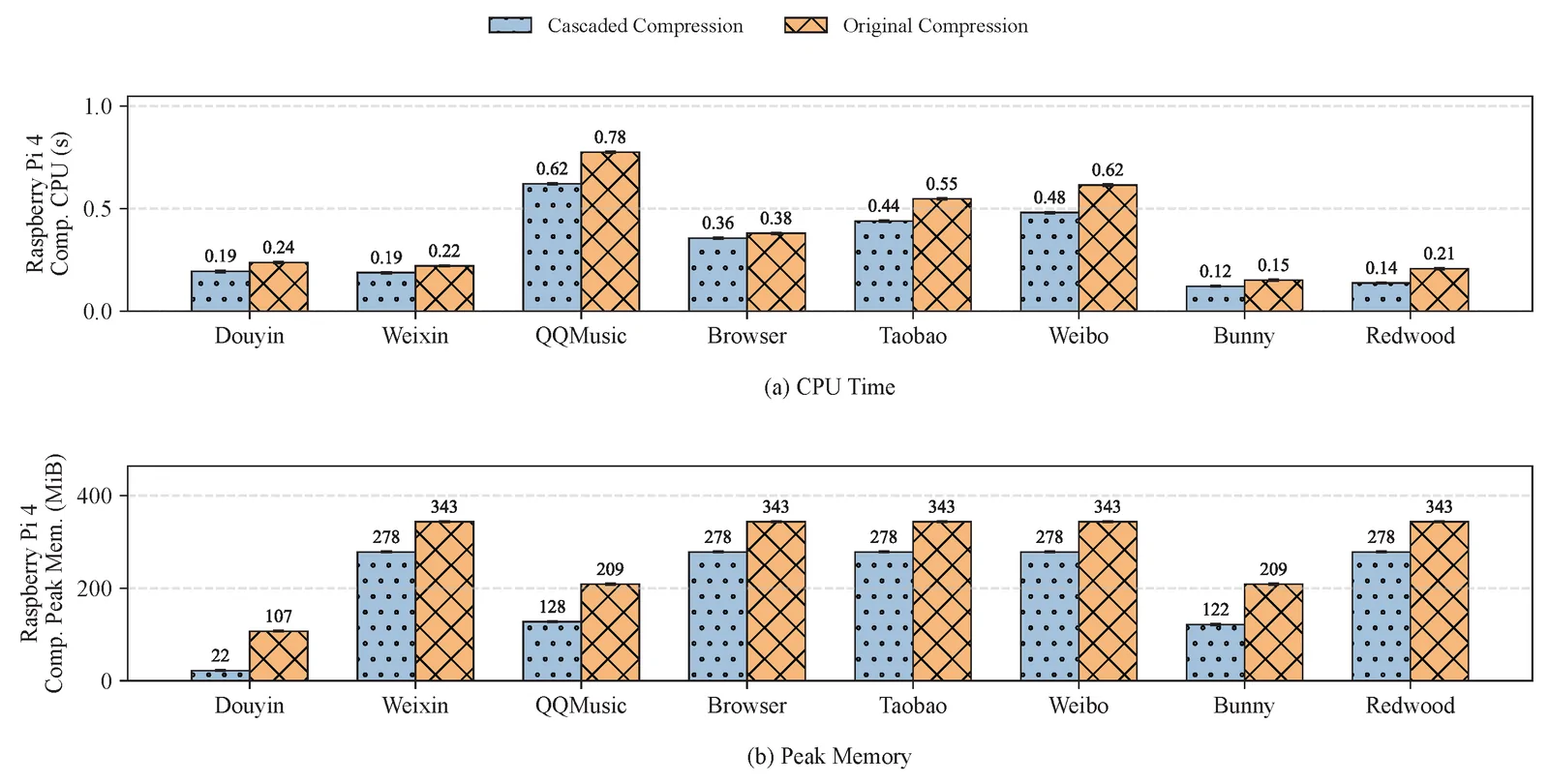
Comparison of resource overhead: (**a**) compression CPU time and (**b**) peak memory usage.

Figure 9 compares the resource overhead of the proposed framework and the restore-then-recompress path. Figure 9a,b report compression CPU time and peak memory usage, respectively. The resource-overhead results are obtained from repeated measurements. For compression-side CPU time, the relative variation is typically below 1%; peak memory usage remains nearly unchanged across runs. The proposed framework incurs substantially lower computational and memory overhead than the conventional path. Specifically, CPU time is reduced by 20.28% on average, with the largest reduction of 33.44% in the Redwood workload. Peak memory usage is reduced by 29.92% on average, with the largest reduction of 79.22% in the Weibo workload. These results indicate that the proposed framework significantly lowers the compute and memory demands of second-stage compression.

The reduction in CPU time mainly comes from avoiding full-page reconstruction and repeated redundancy analysis. The restore-then-recompress path first restores the LZ4-compressed block into the original page and then applies a high-compression algorithm to the reconstructed byte stream, requiring the compressor to rediscover redundancy from the raw page. In contrast, the proposed framework directly reuses the sequence structure generated by LZ4 and performs lightweight transcoding and entropy coding over a structured data stream, thereby reducing CPU time.

The reduction in memory overhead is mainly due to the smaller intermediate representation and the more stable working-set size. The restore-then-recompress path must keep the reconstructed full page and allocate additional workspace for the subsequent high-compression algorithm, increasing runtime memory consumption. In contrast, the proposed framework directly parses, transcodes, and encodes the fields of LZ4 compressed sequences without reconstructing full pages. This design produces smaller intermediate data and yields more stable memory usage during compression.

Overall, by reusing the LZ4 intermediate representation and performing secondary encoding on a compact sequence-level data stream, the proposed framework improves resource efficiency while avoiding full-page reconstruction and repeated compression analysis. It therefore reduces compression latency, CPU time, and peak memory usage, while incurring the compressed-size overhead discussed above.

### 5.4. Decompression Performance and Resource Overhead

The decompression latency and decompression-side resource overhead of the two compression frameworks are further measured. The cascade compression framework does not fundamentally change the decompression path. The entropy-coded payload is still decoded through the same Zstd decompression path as the baseline, and the additional work mainly comes from parsing cascade-block metadata and recovering the LZ4-compatible sequence representation.

**Figure 10:**
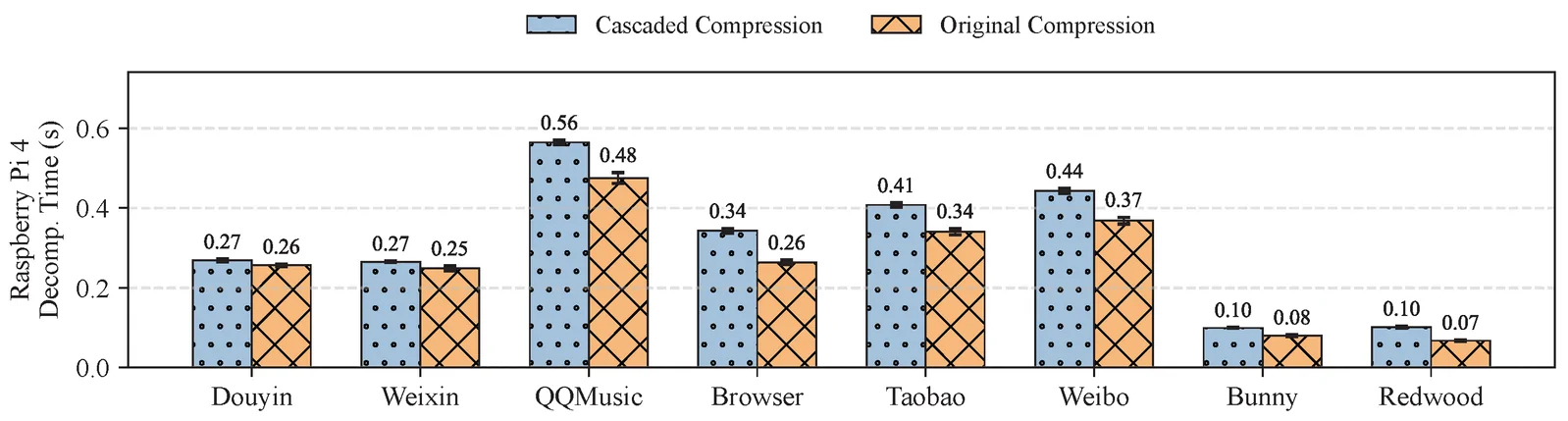
Decompression latency comparison on the Raspberry Pi 4 platform.

As shown in Figure 10, on the Raspberry Pi 4 platform, the decompression latency of the proposed framework remains close to that of the original Zstd path. Across the evaluated workloads, the decompression time of the proposed framework is 1.01–1.51× that of Zstd decompression, with an average of approximately 1.19×. The smallest difference is observed in Weixin, where the ratio is 1.01×, while the largest difference is observed in Redwood, where the ratio reaches 1.51×. These results indicate that the proposed framework introduces only a small additional cost on the decompression side.

**Figure 11:**
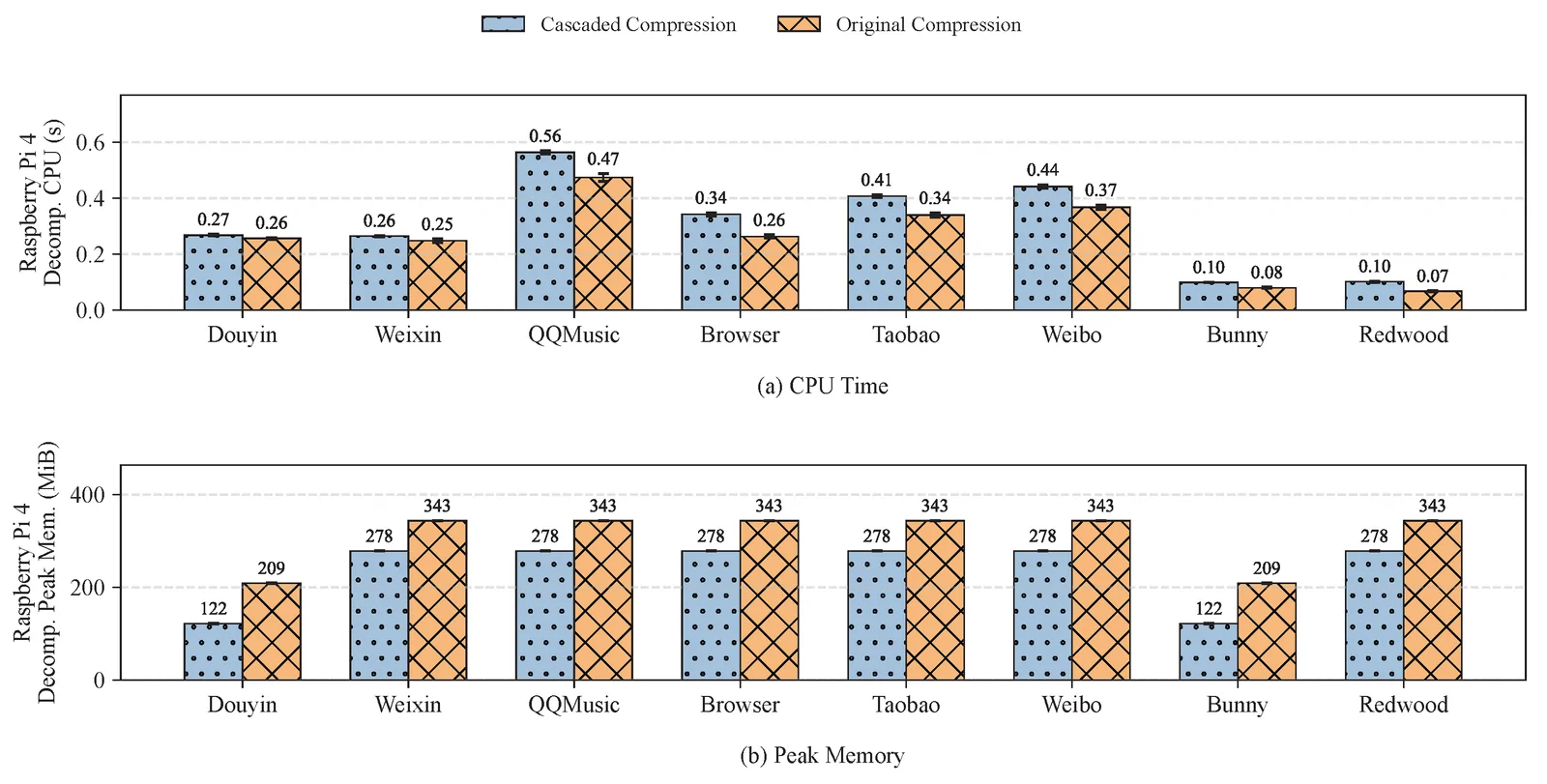
Decompression-side resource overhead on the Raspberry Pi 4 platform: (**a**) CPU time and (**b**) peak memory usage.

Figure 11 further reports the corresponding decompression CPU time and peak memory usage. The trend in CPU time is consistent with the latency results, because decompression follows the same overall decoding path and only adds lightweight metadata parsing and sequence recovery. Peak memory usage remains stable across repeated runs, indicating that the proposed framework does not introduce a large additional decompression working set. Therefore, although the primary benefit of the proposed framework lies on the compression side, the decompression results show that its page-restoration overhead remains low and is still suitable for runtime memory-page recovery scenarios.

### 5.5. Cross-Architecture Validation on RISC-V

To examine whether the above observations depend on a single hardware platform, we conduct an additional evaluation of the proposed framework on a StarFive RISC-V edge platform. We choose RISC-V because its reduced instruction set architecture is increasingly adopted in lightweight and edge-side systems, making it a useful complementary platform to the ARM-based Raspberry Pi 4.

**Figure 12:**
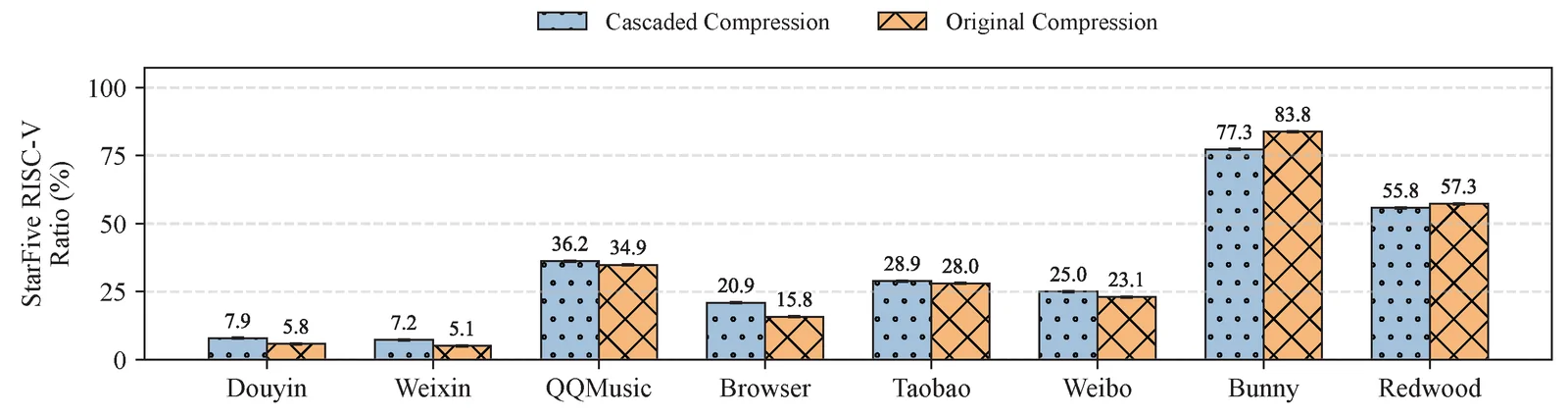
Compressed-size ratio on the StarFive RISC-V platform.

**Figure 13:**
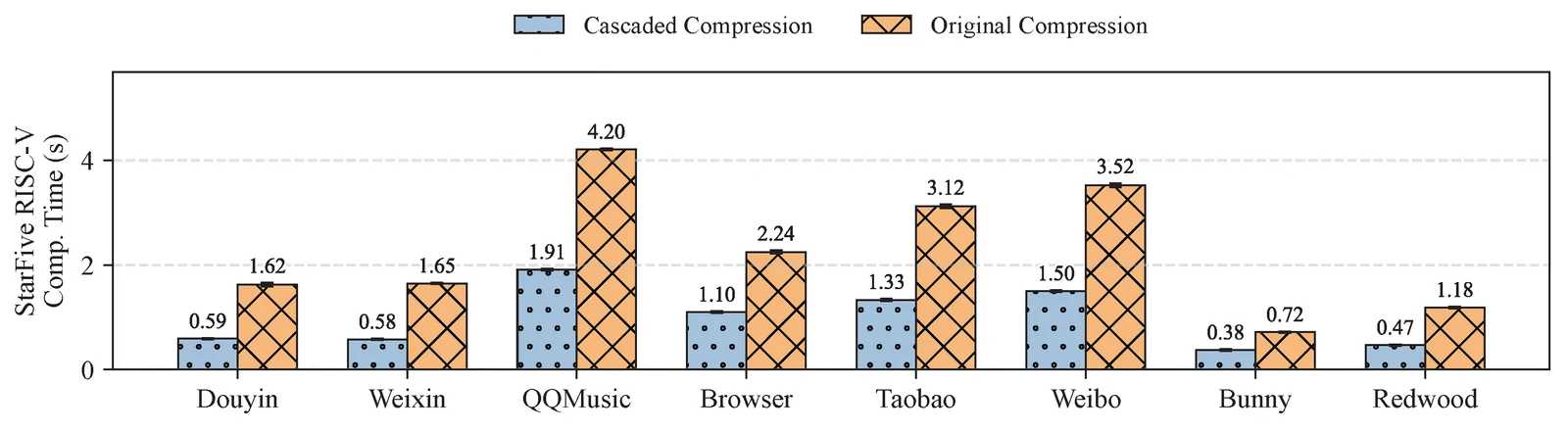
Compression latency on the StarFive RISC-V platform.

As shown in Figure 12 and Figure 13, the results on the RISC-V platform exhibit the same overall trade-off as those on the Raspberry Pi 4 platform. The proposed framework produces higher compressed-size ratios for the six application workloads but lower ratios for the two 3D datasets, while consistently reducing compression latency across all workloads. On the StarFive platform, compression time is reduced by 47.51%–64.36%, with an average reduction of 56.92%.

**Figure 14:**
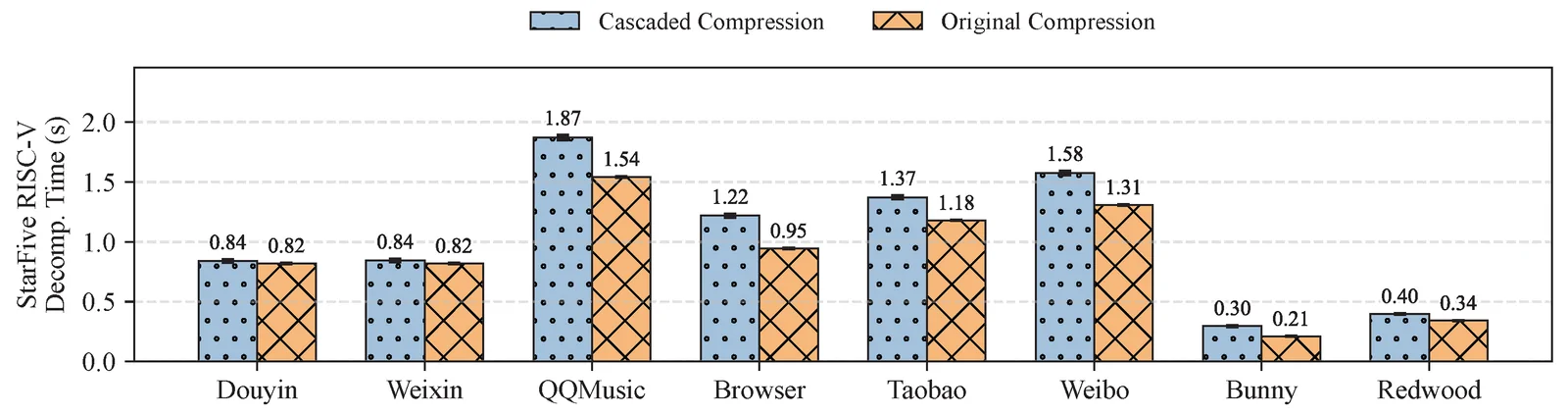
Decompression latency on the StarFive RISC-V platform.

Figure 14 reports the corresponding decompression latency. The decompression time of the proposed framework is 1.04–1.40× that of Zstd decompression, with an average of approximately 1.20×. These results show that the proposed framework maintains the same performance trend on the RISC-V platform as on the Raspberry Pi 4 platform: its main benefit lies in compression-side latency reduction, while the additional decompression overhead remains low.

### 5.6. Functional Correctness and Robustness

This experiment validates whether the proposed framework can preserve byte-level lossless reconstruction and safely handle abnormal inputs. The results show that the proposed framework achieves a 100% reconstruction success rate on more than 200K mixed-entropy memory pages and successfully detects corrupted compressed streams. Specifically, the evaluation covers four representative cases: end-to-end reconstruction, extreme entropy inputs, sequence-boundary cases, and corrupted compressed streams, as summarized in Table 2.

The end-to-end reconstruction test verifies that the proposed framework preserves the original page content and that all tested pages are reconstructed correctly. Each 4-kilobyte page is compressed and then decompressed, and the recovered page is compared with the original input using byte-level comparison. Across more than 200K mixed-entropy pages, all recovered pages are identical to their original inputs.

The extreme entropy test verifies that the proposed framework remains correct under highly skewed data distributions, and all tested extreme inputs are successfully restored. All-zero pages and repeated-byte pages are correctly encoded and decoded without invalid metadata or abnormal decoding behavior.

The sequence-boundary test verifies that field reorganization preserves valid LZ4 sequence semantics, and all tested boundary cases are decoded correctly. The evaluated cases cover long literals, extended match lengths, extreme offsets, and trailing literals, all of which are critical to preserving the recoverability of LZ4 sequences.

The corrupted-stream test verifies that malformed compressed blocks can be safely rejected and that all injected corruptions are successfully detected. Bit-level errors are injected into compressed blocks, and the decoder detects these malformed streams through metadata validation and decoding-state checks.

Overall, these results indicate that the proposed framework preserves lossless reconstruction for the tested pages and can reject malformed compressed streams in the evaluated cases.

### 5.7. Effectiveness of Stream-Specific Entropy Coding

**Figure 15:**
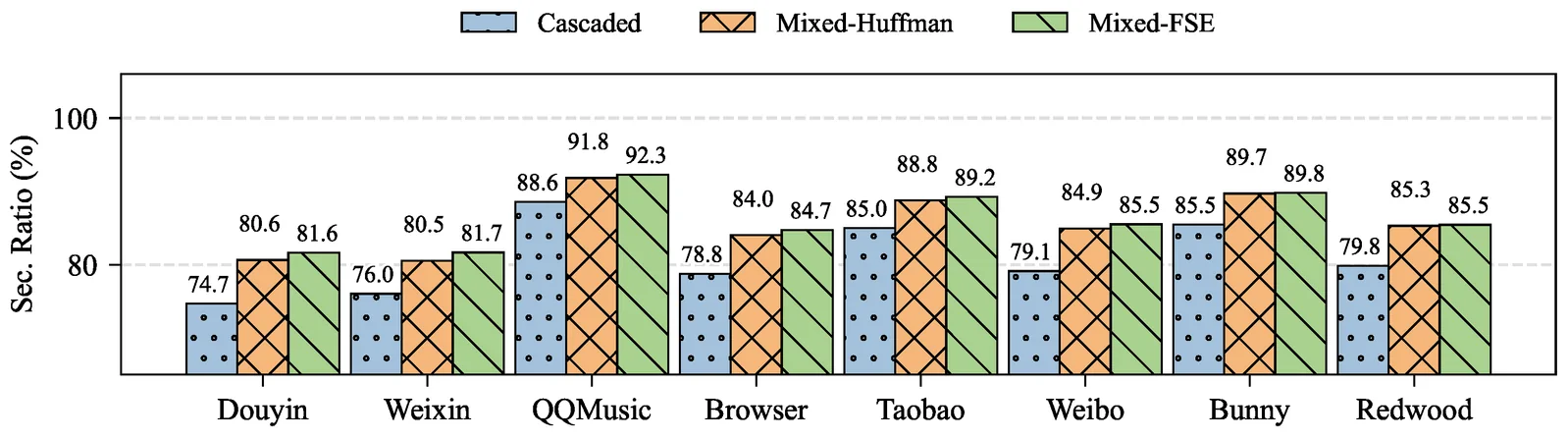
Comparison of secondary entropy-coding strategies.

To validate the effectiveness of the stream-specific entropy-coding strategy, the proposed framework is compared with two uniform coding baselines. Uniform Huffman coding applies Huffman coding to all reorganized streams, while uniform FSE coding applies FSE coding to all streams. As shown in Figure 15, the proposed framework achieves the lowest second-stage compressed-size ratio across all workloads. On average, it reduces the second-stage compressed-size ratio by 4.79 percentage points relative to uniform Huffman coding and by 5.36 percentage points relative to uniform FSE coding. The largest improvement is observed in the Douyin workload, where the reductions reach 6.0 and 6.9 percentage points, respectively.

These results show that using a single entropy coder for all streams is suboptimal. By applying Huffman coding to the literal stream and FSE coding to the sequence streams, the proposed stream-specific strategy better matches the distribution characteristics of different streams and improves secondary compression efficiency.

### 5.8. Effectiveness on 3D Sensing Workloads

This subsection focuses on the results of two representative 16 MiB 3D sensing memory samples: the Stanford Bunny point-cloud sample [35] and the Redwood RGB-D Living Room sample [36]. Their compression-side results are summarized in Table 3.

For the Stanford Bunny sample, the proposed cascaded path reduces the compression time by 33.16% while lowering the compressed-size ratio by 6 percentage points compared with the original restore-then-recompress path. For the Redwood RGB-D Living Room sample, the compression time is reduced by 43.07%, while the compressed-size ratio is lowered by 1.54 percentage points. These results demonstrate the effectiveness of the proposed framework for both point-cloud and real-world RGB-D sensing memory data.

## 6. Discussion

The experimental results show that the proposed sequence-aware cascaded compression framework provides an effective tradeoff for runtime memory compression in edge-side visual and multimedia workloads. Unlike conventional restore-then-recompress schemes, the proposed method does not treat the LZ4 output as an opaque compressed byte stream. Instead, it reuses the sequence-level information already generated by the primary compressor and reorganizes literals, literal lengths, match lengths, and offsets into separated streams. This design changes LZ4 from a standalone fast compressor into a structure-extraction front end for secondary entropy coding. As a result, the second-stage compression process avoids full-page restoration and repeated redundancy analysis, which explains the reductions in processing time, CPU time, and peak memory usage observed in the experiments.

This design is particularly relevant to real-time sensing and edge visual systems. Applications such as 3D visual perception, continuous spatial tracking, augmented interaction, and multimedia analysis continuously generate runtime memory objects, including point clouds, depth maps, image buffers, rendering caches, feature tensors, and media buffers. These data often appear as short-lived memory pages on the system execution path rather than as complete files or frames. Therefore, runtime memory compression must preserve byte-level lossless recovery, introduce low latency, avoid large temporary buffers, and operate under limited CPU and memory budgets. The proposed framework addresses these requirements by compressing page-level runtime data through a lightweight sequence-reuse path rather than a full reconstruction-based recompression path.

The evaluations on the Stanford Bunny and Redwood RGB-D Living Room point-cloud memory samples further demonstrate the applicability of the proposed framework to 3D sensing workloads. For both datasets, the cascaded path reduces compression latency and the compressed-size ratio compared with the conventional restore-then-recompress path. These results show that the sequence information produced during primary LZ4 compression can be effectively reused when processing memory buffers derived from point-cloud and RGB-D data. The stream-specific entropy-coding strategy also remains effective for these structured 3D data buffers, allowing the secondary compression stage to reduce the storage footprint without introducing a complete restoration process.

These properties are valuable for edge-based 3D perception and tracking systems. Point-cloud and RGB-D processing pipelines may retain multiple intermediate buffers for data acquisition, registration, tracking, reconstruction, and visualization. Compressing these buffers with lower processing latency can improve effective memory utilization while limiting the computational overhead introduced by memory management. The current experiments use individual 16 MiB point-cloud memory samples to validate compression-side effectiveness. Further evaluation on continuous RGB-D sequences and complete 3D sensing pipelines is needed to examine sustained throughput, frame-to-frame data variation, and application-level responsiveness.

The proposed method should therefore be understood as a latency- and resource-oriented recompression strategy rather than a maximum-density compressor. Compared with the conventional two-stage recompression baseline, it slightly increases the compressed-size ratio, with an average gap of 0.70 percentage points. However, this limited compression-density loss is accompanied by a 42.10% average reduction in compression processing time, a 20.28% average reduction in CPU time, and a 29.92% average reduction in peak memory usage. For memory-constrained edge devices, such a tradeoff is important because the overhead of compression itself may otherwise offset the benefit of memory saving. In this sense, the proposed framework is more suitable for online runtime memory management than for offline archival compression, where compression density is usually the dominant objective.

The ablation study further confirms the necessity of stream-specific entropy coding. The parsed LZ4 fields exhibit different statistical characteristics: literal bytes are distributed over the byte-value space and show multi-peak behavior, whereas literal lengths, match lengths, and offsets are structured state symbols with head-concentrated and long-tailed distributions. Applying a uniform entropy coder to all streams ignores these differences and weakens compression effectiveness. By assigning Huffman coding to the literal stream and FSE coding to the sequence streams, the proposed framework better matches the statistical properties of each stream. This indicates that the performance gain comes not only from skipping full-page restoration, but also from making the second-stage coding process aware of the semantic structure of the first-stage compression output.

Several system-level issues should also be considered when integrating the proposed framework into practical compressed-memory systems. First, the current evaluation mainly focuses on compression-side latency, CPU time, memory usage, and reconstruction correctness. Although reconstruction correctness has been validated, full-system effects such as decompression latency, page-fault handling, scheduling interference, swap behavior, and application-level responsiveness still require further investigation. Second, although the tested workloads cover representative visual, multimedia, and 3D sensing data, the generality of the method should be further validated on broader sensing workloads, such as robotics perception, augmented reality, video analytics, and embedded 3D reconstruction.

Another integration issue is the cost of dynamic coding decisions. The current implementation adopts fixed stream-specific coding choices, while future systems may dynamically decide whether to apply secondary coding and which coding strategy to use according to page entropy, LZ4 sequence density, literal ratio, stream sizes, or runtime resource pressure. The additional overhead of such dynamic decisions mainly comes from collecting page-level statistics, evaluating policy conditions, and recording small metadata for the selected coding path. This overhead is expected to be modest because many useful statistics, such as sequence count, literal bytes, compressed stream sizes, and LZ4 output size, are already generated during sequence parsing and stream separation. Therefore, the dynamic decision does not require an additional full-page scan or trial recompression before making the coding choice.

The proposed transcoding logic also provides opportunities for hardware-aware optimization on edge devices. The current design can be decomposed into several streaming stages, including LZ4 token parsing, length-extension decoding, literal and offset extraction, LL/ML/OF stream separation, stream packing, and table-based entropy coding. These stages mainly process the compressed block sequentially and pass compact intermediate fields to the next stage, making them suitable for pipeline-style hardware support. For example, a lightweight SoC or FPGA accelerator could parse LZ4 sequence fields in the front-end pipeline, dispatch literals and structured fields into separate buffers, and then feed them to dedicated Huffman/FSE coding units. On ARM-based edge platforms, some byte copying, stream packing, and histogram-related operations may also benefit from NEON/SIMD optimization. Such hardware-aware support could further reduce CPU involvement and improve energy efficiency for real-time edge workloads.

## 7. Concluding Remarks and Future Work

This work presented a sequence-aware cascaded compression framework for runtime memory pages generated by visual and multimedia workloads on edge devices. The key insight is that the output of a fast dictionary compressor can be reused as a structured intermediate representation for secondary entropy coding. Based on this observation, the proposed framework parses LZ4-compressed blocks into literal and structured sequence streams, and applies Huffman and FSE coding according to their statistical characteristics. This avoids the conventional restore-then-recompress path, where the full page must be reconstructed and analyzed again.

The proposed design makes three main contributions. First, it introduces a sequence-aware recompression path that directly reuses LZ4 sequence information, reducing unnecessary data transformation. Second, it separates heterogeneous LZ4 fields into literal and sequence streams, allowing entropy coding to better match their different data distributions. Third, it preserves lossless reconstruction semantics while reducing the computational and memory overhead of secondary recompression.

Experimental results on representative visual and multimedia applications confirm the effectiveness of the proposed framework. Compared with the conventional restore-then-recompress path, the proposed method reduces compression processing time by 42.10% on average and by up to 52.22%, CPU time by 20.28%, and peak memory usage by 29.92%, while maintaining a comparable compressed-size ratio. Functional validation on over 200K mixed-entropy 4-kilobyte pages demonstrates byte-level lossless reconstruction and successful detection of corrupted streams. These results show that the proposed framework reduces the runtime cost of memory compression without sacrificing correctness, making it suitable for memory-constrained edge-side scenarios.

The evaluations on the Stanford Bunny and Redwood RGB-D Living Room point-cloud memory samples further confirm the applicability of the proposed framework to 3D sensing workloads. For both datasets, the cascaded path reduces compression latency and the compressed-size ratio compared with the conventional restore-then-recompress path. These results demonstrate that the proposed sequence-reuse and stream-specific entropy-coding mechanisms can effectively process memory buffers derived from point-cloud and RGB-D data. This capability is particularly relevant to edge-based 3D perception and tracking systems, where large sensing buffers are generated continuously under strict latency, processing, and memory constraints.

Future work will focus on full-system integration and broader validation. First, the framework will be integrated into compressed-memory systems to evaluate decompression latency, page-fault handling, swap behavior, and application responsiveness. Second, lightweight adaptive policies will be investigated to select coding strategies according to page characteristics and runtime resource pressure. Third, decompression-side optimization and hardware-aware acceleration will be further explored on edge platforms. Finally, the evaluation will be extended from individual point-cloud memory samples to continuous RGB-D streams and end-to-end 3D sensing pipelines, including augmented reality, SLAM, robotics vision, and embedded 3D reconstruction, to evaluate sustained processing throughput, frame-to-frame data variation, and system-level effects. 

## Figures and Tables

**Table 1:** Experimental platform configuration.

Item	Raspberry Pi 4	StarFive VisionFive 2
Device	Raspberry Pi 4 Model B	StarFive VisionFive 2
Processor	Broadcom BCM2711, quad-core ARM Cortex-A72	StarFive JH7110, quad-core 64-bit RISC-V U74
Memory	4 GB LPDDR4	8 GB LPDDR4
Storage	MicroSD card, 128 GB	MicroSD card, 128 GB

**Table 2:** Functional correctness and robustness tests.

Test Case	Validation Target	Result
End-to-end reconstruction	Compress and decompress more than 200K mixed-entropy 4-kilobyte pages, followed by byte-level comparison.	100% passed
Extreme entropy inputs	Validate all-zero pages and repeated-byte pages under highly skewed data distributions.	100% passed
Sequence-boundary cases	Validate long literals, extended match lengths, extreme offsets, and trailing literals.	100% passed
Corrupted stream inputs	Inject bit-level errors into compressed blocks to validate malformed-stream detection.	100% detected

**Table 3:** Compression-side results on the 16 MiB 3D sensor memory samples.

Method	Compressed Size (MiB)	Ratio (%)	Total Compression Time (ms)
**Stanford Bunny**			
Original	13.40	83.77	182.30
Cascaded	12.37	77.29	121.86
**Redwood RGB-D Living Room**			
Original	9.17	57.29	243.32
Cascaded	8.92	55.75	138.52

## Data Availability

The data presented in this study are available from the corresponding author upon reasonable request. The raw runtime memory pages are not publicly available due to privacy and security considerations.

## Abbreviations

The following abbreviations are used in this manuscript:

3D	Three-dimensional
ANS	Asymmetric Numeral Systems
BLOSC	Blocking, Shuffling and Compression
CPU	Central Processing Unit
FSE	Finite State Entropy
GZIP	GNU zip
LL	Literal Length
ML	Match Length
OF	Offset

## References

[1] Meng J., Kong Z.J., Hu Y.C., Choi M.G., Lal D. Do we need sophisticated system design for edge-assisted augmented reality?. Proceedings of the 5th International Workshop on Edge Systems, Analytics and Networking.

[2] Gu M., Li K.-C., Li Z., Han Q., Fan W. (2020). Recognition of crop diseases based on depthwise separable convolution in edge computing. Sensors.

[3] Ye Q., Bie H., Li K.-C., Fan X., Gong L., He X. (2022). EdgeLoc: A robust and real-time localization system toward heterogeneous IoT devices. IEEE Internet Things J..

[4] Du K., Zhang Q., Arapin A., Wang H., Xia Z., Jiang J. (2022). AccMPEG: Optimizing video encoding for accurate video analytics. Proc. Mach. Learn. Syst..

[5] Liang W., Xiao J., Chen Y., Yang C., Xie K., Li K.-C., Di Martino B. (2024). Tmhd: Twin-bridge scheduling of multi-heterogeneous dependent tasks for edge computing. Future Gener. Comput. Syst..

[6] Zou G., Qin Z., Deng S., Li K.-C., Gan Y., Zhang B. (2021). Towards the optimality of service instance selection in mobile edge computing. Knowl.-Based Syst..

[7] The Linux Kernel Documentation. zram: Compressed RAM-Based Block Devices. https://docs.kernel.org/admin-guide/blockdev/zram.html.

[8] Li J., Han D., Weng T.-H., Wu H., Li K.-C., Castiglione A. (2025). A secure data storage and sharing scheme for port supply chain based on blockchain and dynamic searchable encryption. Comput. Stand. Interfaces.

[9] Walker L.A., Li Y., McGlothlin M., Cai D. A comparison of lossless compression methods in microscopy data storage applications: Microscopy compression comparison. Proceedings of the 2023 6th International Conference on Software Engineering and Information Management.

[10] Bernstein H.J., Jakoncic J. (2024). Investigation of fast and efficient lossless compression algorithms for macromolecular crystallography experiments. J. Synchrotron Radiat..

[11] Wilson P.R., Kaplan S.F., Smaragdakis Y. The case for compressed caching in virtual memory systems. Proceedings of the 1999 USENIX Annual Technical Conference.

[12] Douglis F. The compression cache: Using on-line compression to extend physical memory. Proceedings of the USENIX Winter 1993 Conference.

[13] Tuduce I.C., Gross T.R. Adaptive main memory compression. Proceedings of the 2005 USENIX Annual Technical Conference.

[14] Collet Y. LZ4 Block Format Description. https://github.com/lz4/lz4/blob/dev/doc/lz4_Block_format.md.

[15] Collet Y., Kucherawy M. Zstandard Compression and the ‘application/zstd’ Media Type. RFC 8878, 2021. https://www.rfc-editor.org/rfc/rfc8878.

[16] Huffman D.A. (1952). A method for the construction of minimum-redundancy codes. Proc. IRE.

[17] Duda J. (2009). Asymmetric numeral systems. arXiv.

[18] Hussain A.J., Al-Fayadh A., Radi N. (2018). Image compression techniques: A survey in lossless and lossy algorithms. Neurocomputing.

[19] Zhang Y., Xiao Y., Zhang Y., Zhang T. (2025). Video saliency prediction via single feature enhancement and temporal recurrence. Eng. Appl. Artif. Intell..

[20] Linardos P., Mohedano E., Nieto J.J., O’Connor N.E., Giro-i-Nieto X., McGuinness K. Simple vs. complex temporal recurrences for video saliency prediction. Proceedings of the 30th British Machine Vision Conference.

[21] Xia C., Zhang M., Gao X., Ge B., Li K.-C., Fang X., Liang X., Zhang Y. (2024). RCFNet: Related cross-level feature network with cascaded self-distillation for monocular depth estimation. Digit. Signal Process..

[22] Wang B., Xia C., Gao X., Yang Y., Li K.-C., Fang X., Zhang Y., Ge S. (2025). Instance-aware sampling and voxel-transformer encoding for single-stage 3D object detection. Digit. Signal Process..

[23] The Linux Kernel Documentation. zswap. https://docs.kernel.org/admin-guide/mm/zswap.html.

[24] Ziv J., Lempel A. (1977). A universal algorithm for sequential data compression. IEEE Trans. Inf. Theory.

[25] Oberhumer M.F.X.J. LZO Real-Time Data Compression Library. https://www.oberhumer.com/opensource/lzo/.

[26] Witten I.H., Neal R.M., Cleary J.G. (1987). Arithmetic coding for data compression. Commun. ACM.

[27] Yang L., Lekatsas H., Dick R.P. High-performance operating system controlled memory compression. Proceedings of the 43rd Annual Design Automation Conference.

[28] Pekhimenko G., Seshadri V., Kim Y., Xin H., Mutlu O., Gibbons P.B., Kozuch M.A., Mowry T.C. Linearly compressed pages: A low-complexity, low-latency main memory compression framework. Proceedings of the 46th Annual IEEE/ACM International Symposium on Microarchitecture.

[29] Choukse E., Erez M., Alameldeen A.R. Compresso: Pragmatic main memory compression. Proceedings of the 51st Annual IEEE/ACM International Symposium on Microarchitecture.

[30] Yang L., Dick R.P., Lekatsas H., Chakradhar S. (2010). Online memory compression for embedded systems. ACM Trans. Embed. Comput. Syst..

[31] Alameldeen A.R., Wood D.A. Adaptive cache compression for high-performance processors. Proceedings of the 31st Annual International Symposium on Computer Architecture.

[32] Hallnor E.G., Reinhardt S.K. A unified compressed memory hierarchy. Proceedings of the 11th International Symposium on High-Performance Computer Architecture.

[33] Shannon C.E. (1948). A mathematical theory of communication. Bell Syst. Tech. J..

[34] Cover T.M., Thomas J.A. (2006). Elements of Information Theory.

[35] Stanford Computer Graphics Laboratory The Stanford 3D Scanning Repository. https://graphics.stanford.edu/data/3Dscanrep/.

[36] Redwood Data. Augmented ICL-NUIM Dataset: Living Room 1. https://www.open3d.org/docs/release/python_api/open3d.data.RedwoodIndoorLivingRoom1.html.

